# Aggressive and Disruptive Behavior Among Psychiatric Patients With Major Depressive Disorder, Schizophrenia, or Alcohol Dependency and the Effect of Depression and Self-Esteem on Aggression

**DOI:** 10.3389/fpsyt.2020.599828

**Published:** 2020-12-03

**Authors:** Michael Fritz, Riad Shenar, Lizbeth Cardenas-Morales, Markus Jäger, Judith Streb, Manuela Dudeck, Irina Franke

**Affiliations:** ^1^Department of Forensic Psychiatry and Psychotherapy, Ulm University, Ulm, Germany; ^2^Department of Psychiatry, Psychotherapy and Psychosomatics, District Hospital Kempten, Kempten, Germany; ^3^Department of Forensic Psychiatry, Psychiatric Services Graubuenden, Cazis, Switzerland

**Keywords:** aggression, depression, schizophrenia, addiction, crime and mental health

## Abstract

Aggressive and disruptive behavior in inpatient settings poses a serious challenge for clinical staff and fellow patients. Hence, the aim of this study was to identify different aspects of aggressive and disruptive behavior in the context of an aberrant self-esteem or clinically manifested depression as potentially influencing factors. We collected self-reported data from 282 psychiatric patients [ICD-10 diagnoses for alcohol dependency, schizophrenia or major depressive disorder (MDD)] and compared it to healthy norm groups. As expected, all three patient groups scored higher in the aggression questionnaires than the norm group. Specifically, patients with MDD exhibited significantly higher externally directed aggression, reactive aggression, and irritability compared to controls. Patients with schizophrenia displayed higher irritability, while all three groups showed distinctly higher self-aggressiveness than healthy persons. We found a lower inhibition of aggression in alcohol dependent subjects compared to both the patient groups and the norm sample. Yet, the higher the self-esteem among alcohol dependent and MDD patients, the lower were their aggression scores; similarly, a lower self-esteem among patients diagnosed with schizophrenia resulted in heighten self-aggressiveness. Thus, our data suggests that therapeutic interventions for strengthening self-esteem in patients with a diagnosis of MDD, alcohol dependency or schizophrenia could reduce certain aspects of aggressive behavior. Therefore, it seems conceivable that strengthening self-esteem in psychiatric patients could contribute to the prevention of violence in clinical practice.

## Introduction

Several studies have highlighted the negative impact of aggressive behavior and violent acts among psychiatric patients in inpatient settings (1, 2). In addition to the serious direct implications of aggression and violence for fellow patients, staff, and environment, frequent disruptive behavior and verbal or physical aggression of patients can genuinely endanger the general goals of psychiatric inpatient care (3).

Although, Hüfner et al. (4) stated that of all reportable work accidents in 2016, violent accidents were proportionally low, about 31% of those occurred among employees in hospitals and nursing homes, psychiatric facilities and emergency rooms. Of notice is though, that the DGUV (Deutsche Gesetzliche Unfallversicherung, German Statutory Accident Insurance) registered an overall increase in the number of this type of accidents since 2016 (5).

The consequence of exposure to patient violence and aggression is psychological stress amongst clinical staff resulting in increased absence time, high internal stress, demoralization, heightened turnover rates, as well as signs of PTSD and a significantly lower level of general health (6–11) together with their colleagues, for instance, reported a higher prevalence of PTSD amongst medical staff in emergency rooms.

Attempts have already been made to identify risk factors for aggressive behavior, e.g., verbal abuse, threats, and physical assault targeted at fellow patients and staff, highlighting a particular role of psychotic patients (1, 12–16). Whereas some authors draw particular attention to the high rates of aggression in inpatients experiencing their first episode of psychosis (17–19) or the effects of comorbid substance use (20, 21), there is an ongoing debate about a broader range of clinical variables and specific patient factors associated with aggression. Such factors are being younger or male, having been admitted involuntarily and having a greater number of previous admissions or a history of violence or self-destructive behavior or both (22). Other studies demonstrated that an exacerbation of psychotic symptomatology (e.g., persecutory ideations, delusions, hallucinations, excitement, poor impulse control, and thought disturbances) was associated with increased risk of violent behavior on the ward (23, 24). Rund (25) showed that insight, impulsivity, psychopathy, motor speed and a global measure of cognition have strong empirical evidence for an association with violence.

Substantial evidence demonstrates a link between drug dependence and aggressive behavior in various patient groups: Bernstein et al. (26), for instance, found significantly higher impulsivity among imprisoned lifetime drinkers. Hoaken and Stewart (27) reviewed the existing literature and stated that there is clear evidence to support the thesis of a direct alcohol intoxication-violence relationship compared to other drugs of abuse. The meta-analysis of Iozzino et al. (28) examined data of 23,972 psychiatric inpatients and showed that patients with alcohol use disorder reported higher rates of inpatient violence. Rund (25) showed that substance abuse is robustly linking schizophrenia and violence.

On the other hand, depressive symptoms were initially considered to be slightly protective against episodes of aggression on inpatient wards (29). Such a point of view seems reasonable, particularly with regard to the inhibiting characteristics of depression, but neglects the self-aggressive features of depressive symptoms. Brugman et al. (30) performed a study in a non-clinical, male sample and found that stronger self-aggression was associated with more aggressive behavior under experimental conditions. Similar results are reported by Dudeck et al. (31). Also, a recent Swedish population study conducted by Fazel et al. (32) indicated that the risk of violent crime was significantly greater in individuals with an index diagnosis of depression, even after adjustment for familial, sociodemographic, and individual factors. Salem et al. (33) showed that anhedonia may explain the relationship between depression and aggressive and antisocial actions.

The connection between self-esteem and aggressive behavior has been debated in various studies [see (34–39)]. Ostrowsky (40) reviewed the literature about the association between self-esteem and aggression and concluded it to inconsistent. This may be because research on self-esteem (e.g., global self-esteem vs. different dimensions of self-esteem, high vs. low self-esteem, stable vs. unstable self-esteem, narcissism) and aggression (e.g., verbal vs. physical violence, impulsive vs. reactive aggression, a mediating role of victimization, etc.) has focused on various concepts, subtypes, and combinations of these [see Chung et al. (41)]. Otte et al. (42) showed that different levels of self-esteem influence various forms of aggression in people from the general population and psychiatric patients. For instance, high self-esteem seems to prevent self-aggression, but increases the probability for the occurrence of reactive and spontaneous aggression in both groups. This may suggest that self-aggression is a copying strategy to escape aversive internal states of shame, self-punishment or rejection—internal states a person with high self-esteem rarely encounters (42).

However, the literature is sparse on the association between self-esteem and aggression in specific psychiatric diagnoses such as schizophrenia, MDD, or alcohol dependency. Garthe and Schacht (43) found that low self-esteem and covert aggression were correlated with depression in Scottish college students. Yet, the connection in adults diagnosed with schizophrenia, MDD, and alcohol dependency remains unclear.

Few studies have directly compared aggression on psychiatric inpatient wards in different disorders and the moderating role of self-esteem and depression. Therefore, the aim of the present study was to identify differences in aggressiveness factors, aspects of aggressive and disruptive behavior, and willingness to act aggressively and the role of multidimensional self-esteem and depression in three common diagnostic groups of patients on psychiatric wards in Germany.

## Methods

### Ethical Approval Statement

The study was conducted in accordance to the Declaration of Helsinki and approved by the ethical review committee of the University of Ulm (No. 173/14).

### Participants and Procedure

The basis of the present study was a cross-sectional data collection conducted in two hospitals (Department of Psychiatry and Psychotherapy, Helios Hanseklinikum, Stralsund; Department of Psychiatry and Psychotherapy II, Ulm University, Günzburg) Germany. The total sample consisted of psychiatric patients (*N* = 282; female, *n* = 136) who were receiving treatment (inpatient, *n* = 159; day-hospital care, *n* = 108, and outpatient, *n* = 15). Patients were diagnosed by experienced clinicians on the basis of the ICD-10 criteria and separated into the following groups: (1) alcohol dependency (*n* = 92), (2) schizophrenia (*n* = 51), and (3) MDD (*n* = 139). These three diagnoses were preselected, as they were the three most common ones in both department hospitals. Exclusion criteria were insufficient language skills, intellectual disability, or neurological disorders. Special care was taken to ensure that there was no comorbidity between the diagnostic groups. After signing informed consent, participants completed a set of questionnaires.

### Measures

In addition to collecting demographic information, the following self-assessment measures were applied:

#### Aggressiveness Factors

To measure aggressiveness factors, aspects of aggressive behavior, and willingness to act aggressively, participants completed a German questionnaire, the Kurzfragebogen zur Erfassung von Aggressivitätsfaktoren [K-FAF, (44)], which is conceptually based on models of the learning psychology of aggressive behavior. The K-FAF, a 49-item self-report scale, is the revised, shorter version of the FAF (45). Participants were asked to rate the statements on a 6-point scale from 0 (strongly disagree) to 5 (strongly agree). The questionnaire consists of five subscales: (1) impulsive aggression, (2) reactive aggression, (3) irritability, (4) self-aggressiveness, and (5) inhibition of aggression. The impulsive aggression scale asks about imagined, verbal, and physical aggression against people and animals and also about sadistic tendencies (e.g., “Sometimes I like to torment other people”). Reactive aggression depicts societally sanctioned aggressive behavior, high scores indicate high assertiveness, and low scores suggest disapproval of aggressive and disruptive behavior styles (e.g., “If I'm treated badly, I want revenge.”). High values on the irritability scale increasingly reflect anger, as well as a lack of emotion regulation and a low tolerance for frustration (e.g., “I lose my temper quickly, but calm down quickly, too”). The self-aggression scale comprises items on self-reproach, resentment, mistrust, depressive mood, and suicidal thoughts; it thus focuses on more subtle forms of self-aggression than self-injury or suicide and rather represents a mental form of self-aggression (e.g., “I have seriously considered suicide”). High values on the scale of inhibition of aggression should express knowledge of common norms. Subscales 1–3 can be combined as the summary scale “sum of aggression,” which represents the potential for externally directed aggression.

#### Multidimensional Self-Esteem

To assess multidimensional self-esteem, participants completed the German version of the Multidimensionale Selbstwertskala [MSWS, (46)], adapted from the Multidimensional Self-Concept Scale (MSCS, Fleming and Courtney, 1984). The MSWS is a 32-item self-report measure that captures global self-esteem as well as various individual aspects of self-esteem (e.g., emotional self-esteem, performance-related self-esteem, social self-esteem regarding dealing with criticism and self-esteem regarding physical attractiveness). Items are answered on a 7-point scale ranging from “not at all” to “strongly” and from “never” to “always.” Because of the hierarchical structure of the questionnaire, subscales can be assigned to two summary scales (general self-esteem and physical self-esteem), which in turn can be combined to give a rating of total self-esteem.

#### Depression

The Beck Depression Inventory-II [BDI-II, (47–49)] was used to assess symptoms and severity of depression and corresponding behavior. The BDI-II is one of the most commonly and frequently used self-rating scales to evaluate the severity of depression (50) and measures depressive symptoms via 21 items, with a focus on the past 2 weeks to the present day. Statements are rated on a 4-point scale, ranging from 0 to 3. Total scores range from 0 to 63 and indicate different degrees of severity and numbers of symptoms, depending on recommended cut-off scores.

### Statistical Analysis

Three-way ANCOVA analysis was conducted with the dependent variables being the sum of aggression, self-esteem, depression and all aggression subscales. The three factors were “patient group,” “gender,” and “treatment” (=inpatient, day-hospital care/outpatient), whereas “age” was considered as a covariate. The significant main effects of patient group were further analyzed by Bonferroni-corrected *post hoc* comparisons.

For the statistical analyses, we used the norm samples specified in the measures' relevant manuals as a healthy control group. The K-FAF manual provides a norm sample (*N* = 397) consisting of people aged 18–75 years with no criminal record (44). The norm sample of the MSWS (*N* = 453) was stratified by age (14–92 years) and gender to be as representative of the population as possible (46). The BDI-II features a healthy norm sample (*N* = 582) of the general population with a mean age of 39 years (47). We used *t*-tests for independent samples (https://www.medcalc.org/calc/comparison_of_means.php) to compare all mean values with the measure's particular norm samples.

Finally, we calculated Pearson correlations between the aggression scales of the K-FAF and the self-esteem scale (MSWS), for the total sample and separately for the three patient groups.

## Results

### Socio-Demographic Data

Regarding socio-demographic data the patient groups differed regarding age, gender, and treatment, and analyses were controlled for those variables to exclude confounding effects (see Table 1). They also differed regarding family status and current occupation. The three patient groups did not differ regarding their educational level.

**Table 1:** Demographic information.

	**Alcohol dependency**	**Schizophrenia**	**Major depression**	***F*(df1,df2)*/χ*^2^(df)/ETF**
	*n* = 92	*n* = 51	*n* = 139	
	*M* (*SD)/n* (%)	*M* (*SD)/n* (%)	*M* (*SD)/n* (%)	
Age	46.63 (10.87)	38.68 (12.47)	48.38 (9.62)	15.836**(2,279)^a^
Gender				
Male	69 (75)	29 (57)	48 (35)	36.955**(2)^b^
Female	23 (25)	22 (43)	91 (66)	
Family status				
Single	28 (30)	34 (67)	17 (12)	69.278**(6)^b^
Married/Partnership	26 (28)	9 (18)	79 (57)	
Divorced	36 (39)	8 (16)	36 (26)	
Widowed	2 (2)	0 (0)	7 (5)	
Educational level				
No educational qualification	6 (7)	5 (10)	13 (9)	6.269 (4)^b^
Secondary school (10 years' schooling)	74 (80)	39 (77)	100 (72)	
High school (12 years' schooling)	12 (13)	7 (14)	26 (19)	
Current occupation				50.017** (8)^b^
Unemployed	54 (59)	19 (37)	32 (23)	
Employed	18 (20)	5 (10)	58 (42)	
Pensioner	18 (20)	24 (47)	47 (34)	
Student	1 (1)	2 (4)	0 (0)	
Vocational training	1 (1)	1 (2)	2 (1)	
Treatment				57.534**^c^
Outpatients	11 (12)	0	4 (3)	
Day-hospital care	47 (51)	2 (4)	59 (42)	
Inpatients	34 (37)	49 (96)	76 (55)	

a *Value of F-score*,

b *Value of Pearson's Chi-square χ^2^*,

c *Value of Fisher's exact test*,

** *p < 0.01*.

### Main Scales

In a first approach, the main scales of the applied measures were analyzed by three-way ANCOVA with the factors patient group, gender, and treatment and by controlling for age (Table 2).

**Table 2:** Two-way ANCOVA results of main scales and Bonferroni-corrected *post hoc* tests.

	***F*-statistics**	***Post hoc* comparisons**
DV: Sum of aggression		
Treatment	*F*_(1, 275)_ = 0.120	
Group	*F*_(2, 275)_ = 2.313	
Gender	*F*_(1, 275)_ = 2.142	
Group x Gender	*F*_(2, 275)_ = 0.977	
DV: Total self-esteem		
Treatment	*F*_(1, 275)_ = 0.020	
Group	*F*_(2, 275)_ = 13.506**	Major depression > Alcohol dependency**, Major depression > Schizophrenia*
Gender	*F*_(1, 275)_ = 6.271*	Women > Men**
Group x Gender	*F*_(2, 269)_ = 0.075	
DV: BDI score		
Treatment	*F*_(1, 275)_ = 4.493	
Group	*F*_(2, 275)_ = 31.297**	Major depression > Alcohol dependency**, Major depression > Schizophrenia**
Gender	*F*_(1, 275)_ = 6.660*	Women > Men*
Group x Gender	*F*_(2, 275)_ = 0.907	

DV, dependent variable, Age was considered as covariate in all analyses, *p < 0.05, **p < 0.01.

#### Sum of Aggression

The three-way ANCOVA regarding externally directed aggression (“sum of aggression”) found no significant differences among patient groups, treatment or between genders and no significant interaction between patient group and gender (see Table 2). However, the *t*-test showed that the group of patients with MDD had significantly higher scores for externally directed aggression than the measure's norm sample (see Table 3).

**Table 3:** *t*-test results of main scales.

	**Patients**	**Norm sample**	
	***M (SD)***	***M (SD)***	***t*** **(df)**
DV: Sum of aggression			
Alcohol dependency	44.43 (28.16)	43.42 (17.30)	0.44 (487)
Schizophrenia	49.12 (26.62)	43.42 (17.30)	2.76 (446)
Major depression	49.26 (25.04)	43.42 (17.30)	3.02* (534)
DV: Total self-esteem			
Alcohol dependency			
Male	158.06 (38.24)	165.61 (23.27)	−1.94** (268)
Female	147.17 (32.94)	151.68 (30.30)	−0.673* (235)
Schizophrenia			
Male	148.76 (38.36)	165.61 (23.27)	−3.31** (228)
Female	132.59 (36.76)	151.68 (30.30)	−2.76** (234)
Major depression			
Male	129.60 (32.18)	165.61 (23.27)	−8.89** (247)
Female	117.46 (41.72)	151.68 (30.30)	−8.02** (303)
DV: BDI score			
Alcohol dependency	14.26 (11.67)	7.4 (7.3)	7.61** (672)
Schizophrenia	17.73 (10.25)	7.4 (7.3)	9.34** (631)
Major depression	27.32 (10.57)	7.4 (7.3)	26.27** (719)

*p < 0.05, **p < 0.01.

#### Total Self-Esteem

For total self-esteem, the three-way ANCOVA showed significant group differences among the patient groups and significant differences between men and women. Patients with MDD had higher scores than those with alcohol dependency or schizophrenia, and women had higher scores than men. There was neither a significant main effect for treatment nor a significant interaction between patient group and gender (see Table 2). In *t*-tests, total self-esteem scores were significantly lower in all three patient groups than the measure's respective norm sample (see Table 3).

#### Depression (BDI Score)

The three-way ANCOVA for the BDI score revealed significant group differences within the group of patients and between the genders. Again, patients with MDD had higher scores than patients with alcohol dependency or schizophrenia, and women had higher scores than men. There was neither a significant main effect for treatment nor a significant interaction between patient group and gender (see Table 2). The *t*-tests showed that all three patient groups achieved significantly higher depression scores than the respective mentally healthy norm sample of the BDI (see Table 3).

### Aggression (K-FAF Subscales)

Further results for the subscales impulsive aggression, reactive aggression, irritability, self-aggressiveness, and inhibition of aggression revealed various differences among patient groups, which are presented in Table 4.

**Table 4:** Two-way ANCOVA results of the Kurzfragebogen zur Erfassung von Aggressivitätsfaktoren (K-FAF) subscales and Bonferroni-corrected *post hoc-*tests.

	***F*-statistics**	***Post hoc* comparisons**
DV: Impulsive aggression		
Treatment	*F*_(1, 275)_ = 0.046	
Group	*F*_(2, 275)_ = 2.157	
Gender	*F*_(1, 275)_ = 6.705*	Male > Female*
Group x Gender	*F*_(2, 272)_ = 0.252	
DV: Reactive aggression		
Treatment	*F*_(1, 275)_ = 0.057	
Group	*F*_(2, 275)_ = 1.503	
Gender	*F*_(1, 275)_ = 3.359	
Group x Gender	*F*_(2, 275)_ = 1.110	
DV: Irritability		
Treatment	*F*_(1, 275)_ = 0.014	
Group	*F*_(2, 275)_ = 2.793*	Major depression > Alcohol dependency*
Gender	*F*_(1, 275)_ = 0.032	
Group x Gender	*F*_(2, 275)_ = 2.891*	Schizophrenia x Female > Schizophrenia x Male*
DV: Self-aggressiveness		
Treatment	*F*_(1, 275)_ = 3.440	
Group	*F*_(2, 275)_ = 9.229**	Major depression > Alcohol dependency**; Major depression > Schizophrenia*; Schizophrenia > Alcohol dependency*
Gender	*F*_(1, 275)_ = 0.754	
Group x Gender	*F*_(2, 275)_ = 1.362	
DV: Aggression inhibition		
Treatment	*F*_(1, 275)_ = 0.173	
Group	*F*_(2, 275)_ = 3.469*	Major depression > Alcohol dependency*, Schizophrenia > Alcohol dependency*
Gender	*F*_(1, 275)_ = 0.003	
Group x Gender	*F*_(2, 275)_ = 3.081*	Alcohol dependency x Male > Alcohol dependency x Female*

Age was considered as covariate in all analyses, DV, dependent variable, *p < 0.05, **p < 0.01.

#### Impulsive Aggression

Impulsive aggression did not differ significantly among the three patient groups and between treatments, but scores were higher in men than in women. There was no significant interaction between patient group and gender (see Table 4). The alcohol-dependency group showed lower impulsive aggression scores than the measure's norm sample (*t*_[487]_ = −2.17, *p* < 0.05).

#### Reactive Aggression

Reactive aggression also did not differ among the patient groups, treatment or between men and women, and there was no significant interaction between patient group and gender (see Table 4). However, the group of patients with MDD showed significantly higher reactive aggression scores than the measure's norm sample (*t*_(534)_ = 2.22, *p* < 0.05).

#### Irritability

The analysis of irritability revealed significant group differences among patient groups but no differences between men and women or between treatments. *Post hoc* comparisons showed that patients with MDD had higher scores than patients with alcohol dependency. In addition, there was a significant interaction between patient group and gender for patients with schizophrenia, with higher scores of irritability in women than in men (see Table 4). Furthermore, patients with MDD (*t*_(534)_ = 4.39, *p* < 0.01) and those with schizophrenia (*t*_(446)_ = 2.29, *p* < 0.05) showed significantly higher irritability scores than the measure's norm sample.

#### Self-Aggressiveness

The ANCOVA for self-aggressiveness showed significant differences between the patient groups, but neither gender-related differences, treatment-related differences nor the interaction between patient group and gender was significant (see Table 4). *t*-tests showed that all three patient groups had significantly higher self-aggressiveness than the measure's norm sample: alcohol dependency (*t*_(487)_ = 6.01, *p* < 0.01), schizophrenia (*t*_(446)_ = 7.46, *p* < 0.01), and MDD (*t*_(534)_ = 18.12, *p* < 0.01).

#### Inhibition of Aggression

The ANCOVA regarding the inhibition of aggression found significant differences among the patient groups but no significant discrepancy between men and women or among treatment. Bonferroni-corrected *post hoc* tests showed that patients diagnosed with alcohol dependency had significantly lower scores for inhibition of aggression than patients with schizophrenia or MDD. There was a significant interaction between patient groups and gender. *Post hoc* comparisons showed that men with alcohol dependency had higher scores than women (see Table 4). *t*-tests showed that alcohol dependent patients had significantly lower inhibition of aggression than the measure's norm sample (*t*_(487)_ = 4.59, *p* < 0.01).

### Subscales of Aggression and Self-Esteem

Pearson's correlations between the subscales of aggression and self-esteem were calculated for the whole patient sample and separately for all patient groups. All in all self-esteem correlated negatively with all aggressiveness factors (see Table 5).

**Table 5:** Correlations between the subscales of the Kurzfragebogen zur Erfassung von Aggressivitätsfaktoren (K-FAF) and self-esteem (MSWS).

	**Self-esteem**	
	***r***	***p***
Impulsive aggression	−0.293	<0.001
Reactive aggression	−0.243	<0.001
Irritability	−0.261	<0.001
Self-aggressiveness	−0.720	<0.001
Aggression inhibition	−0.272	<0.001
Sum of aggression	−0.306	<0.001

*Age was considered as covariate*.

In the subgroups of patients with alcohol dependency and MDD, self-esteem and aggression correlated significantly negatively. For the schizophrenic patients there was no correlation between self-esteem and externalizing aggression, but self-esteem correlated significantly negatively with self-aggressiveness (see Table 6).

**Table 6:** Pearson correlations between the subscales of the Kurzfragebogen zur Erfassung von Aggressivitätsfaktoren (K-FAF) and self-esteem (MSWS).

	**Self-esteem**		
	**Alcohol dependency *r***	**Major depression *r***	**Schizophrenia *r***
Impulsive aggression	−0.535**	−0.236**	−0.038
Reactive aggression	−0.367**	−0.229**	−0.081
Irritability	−0.264**	−0.243**	−0.189
Self-aggressiveness	−0.716**	−0.705**	−0.640**
Aggression inhibition	−0.277**	−0.317**	−0.006
Sum of aggression	−0.416**	−0.278**	−0.131

Age was considered as covariate, **p < 0.01.

## Discussion

Our findings indicate that patients with MDD, schizophrenia, or alcohol dependency did not differ regarding externally directed aggression when analyses were controlled for age and gender. However, patients with MDD seemed to show higher externally directed aggression than the respective healthy norm sample. These findings are in line with the results of two Swedish longitudinal studies reporting an increased risk of violent crimes in individuals with depression (32). Here, the authors found an odds ratio for violent crime of 3.0 (95% CI 2.8–3.3) compared to general population controls after adjustment for sociodemographic confounders. The increased odds remained significant in depressed persons after adjustment for familial confounding, substance abuse comorbidity, and history of previous violent convictions.

Furthermore, our findings elucidate insufficiencies regarding total self-esteem among all three patient groups. First, MDD patients scored significantly higher in total self-esteem than the other two groups. Secondly, higher self-esteem in alcohol dependent MDD patients correlated with lower the scores of aggressiveness and vice versa and thirdly lower self-esteem in schizophrenic patients related to higher scores of self-aggressiveness and vice versa.

Depression scores assessed by the BDI, were increased in all three patient groups compared to the healthy norm sample, with MDD patients scoring significantly higher than the others.

Particularly in terms of self-aggressiveness, patients with MDD appear to be not only more outgoing aggressive than a healthy norm sample, but also toward themselves than patients with schizophrenia or alcohol dependency. These findings are in line with results regarding suicidal ideation (51) and with those regarding completed suicides in men diagnosed with MDD and higher levels of aggression (52).

Moreover, patients with major depression or schizophrenia had significantly higher irritability scores than a healthy norm sample. While female psychotic patients seemed to be more irritable than male psychotic patients, no gender differences were found among patients with MDD. These results indicate more anger- and rage-related experiences, low frustration tolerance, and a lack of affect regulation among patients with MDD. Especially, the lack of affect can be expected in affective disorders.

Scores for reactive aggression were also higher in the sample of MDD patients compared to the norm sample. These results suggest greater and more determined assertiveness among depressive patients, even though they present specific depressive symptoms. However, Rezayat and Nayeri (53) report an inverse correlation between depression (also measured by the BDI) and assertiveness among a sample of nursing students. These opposing results may be due to the varying life circumstances of inpatients and nursing students. The actual role of self-esteem might become particularly important in this context. Our findings on the role of self-esteem and aggression seem to support previous findings of self-esteem as a suppressor variable on different aspects of aggression as shown by Otte et al. (42).

Regarding impulsive aggression, alcohol-dependent patients described themselves as more restrained and calm or even passive when rating fantasized, verbal, or physical aggression against other humans or animals. On the other hand, our findings suggest significantly lower scores for inhibition of aggression in alcohol-dependent patients than in a healthy norm sample and even than in patients with schizophrenia or MDD. These findings are in line with a study by Hwang et al. (54), which showed higher levels of impulsiveness and anger expression among help-seeking alcohol-dependent patients than healthy controls. Also, a review of neurocognitive research results conducted by Stevens et al. (55) supports an important role of cognitive disinhibition, delay discounting, and impulsive decision-making in the ability to achieve abstinence during addiction treatment.

To our knowledge, this is the first study comparing aggressive behavior among three common diagnostic groups of psychiatric patients. Furthermore, aggression was not only considered in terms of potential violent acts against staff or fellow patients or both, but also in terms of self-aggressiveness, dissatisfaction, and a negative attitude toward life. Moreover, our findings demonstrate that self-aggressiveness has the strongest inverse correlation with self-esteem and hence might be considered a robust predictor for suicidality.

However, there are some limitations to this work. The results should be considered with caution concerning self-report measures, as they may be subject to social desirability bias and errors in self-observation. In fact, neither the amount nor the frequency of objectively accountable aggressive incidences were recorded to cooperate self-reports. Secondly, the sample includes data from outpatients, who may have a lower severity of symptoms, which could have influenced the results of the present study. It should be noted that some patients, particularly schizophrenic patients, may have misinterpreted or misunderstood self-assessment questions about self-esteem and may have affected the results of the study because it is conceivable that these patients cannot understand the concept of self-esteem due to their illness. Hence, it would have been useful to assess the momentary psychotic symptomatology present in schizophrenia and sometimes also in major depressive disorder with for example the PANS-scale, while conducting the interview. Furthermore, measured self-esteem values may be affected by symptoms of acute mental illness. In our study no information on medication is provided. Because psychiatric medication can affect aggression, this could have confounded our results.

Conclusively, our results indicated that patients diagnosed with MDD exhibited significantly higher externally directed aggression, reactive aggression, and irritability than a healthy norm sample. Moreover patients diagnosed with schizophrenia also showed higher irritability than the norm sample, while all three groups showed distinctly heightened self-aggressiveness when compared to healthy persons. Patients diagnosed with alcohol dependency, however, exhibited lower inhibition of aggression than the other patient groups and the healthy norm sample. The level of self-esteem showed a negative correlation to all factors of aggressiveness in patients with MDD and alcohol dependency and may therefore support therapeutic interventions for improvement of self-esteem in these patient groups. It therefore seems conceivable that an improvement in self-esteem in the examined patient groups could result in a reduction of aggressive behavior. In view of the increasing number of aggressive and disruptive behavior by patients in medical facilities, the contemplated considerations for therapeutic interventions could contribute to the prevention of violence in clinical practice.

Further research should include behavioral observations and observer rating measures. Furthermore, patients should be compared with forensic samples.

## Data Availability Statement

The raw data supporting the conclusions of this article will be made available by the authors, without undue reservation.

## Ethics Statement

The studies involving human participants were reviewed and approved by Ethical Review Committee of the University of Ulm (No. 173/14). The patients/participants provided their written informed consent to participate in this study.

## Author Contributions

MF and RS together with LC-M, JS, MD, and IF wrote the manuscript. RS collected the data and analyzed them with assistance of JS. MJ, MD, and IF provided crucial input to the study design. All authors read, consented and commended on the manuscript.

## Conflict of Interest

The authors declare that the research was conducted in the absence of any commercial or financial relationships that could be construed as a potential conflict of interest.
